# Hematologists/Physicians Need to Be Aware of Pseudohypercalcemia in Monoclonal Gammopathy: Lessons from a Case Report

**DOI:** 10.1155/2024/8844335

**Published:** 2024-08-19

**Authors:** Svenja F. B. J. Mennens, Ellen Van der Spek, Janneke Ruinemans-Koerts, Marcel M. G. J. Van Borren

**Affiliations:** ^1^ Laboratory of Clinical Chemistry and Hematology Rijnstate Hospital, Arnhem, Netherlands; ^2^ Department of Clinical Chemistry DICOON BV, Arnhem, Netherlands; ^3^ Department of Internal Medicine Rijnstate Hospital, Arnhem, Netherlands

## Abstract

We present a patient at risk of misdiagnosis with multiple myeloma due to pseudohypercalcemia. Examinations showed monoclonal protein, 50% monoclonal plasma cells in bone marrow, and hypercalcemia but no osteolytic bone lesions. Follow-up tests revealed pseudohypercalcemia, with elevated total calcium, but normal ionized calcium: a discrepancy due to calcium binding to monoclonal paraprotein (confirmed by laboratory experiments). Accordingly, the patient was diagnosed with smouldering myeloma. After 900 days, the presence of bone lesions prompted the start of treatment for myeloma. Consequently, monoclonal paraprotein levels declined and pseudohypercalcemia dissolved. Hence, ionized calcium should be measured in monoclonal gammopathies to avoid misdiagnosis.

## 1. Introduction

Multiple myeloma is a hematological proliferative disorder based on clonal expansion of plasma cells [1]. It is important to distinguish multiple myeloma (requiring therapy) from MGUS (monoclonal gammopathy of undetermined significance) and smouldering myeloma. The latter two do not necessarily require therapy but represent a premalignant and intermediate clinical stage, respectively, with increasing risk of progression to malignant disease [1]. To classify as multiple myeloma (in contrast to MGUS or smouldering myeloma), the presence of one of the myeloma-defining events is required (i.e., CRAB criteria: elevated calcium; renal failure; anemia; and bone lesions) [1]. Hypercalcemia as a result of osteolytic bone lesions is a typical hallmark of multiple myeloma. However, in rare cases, plasma calcium is falsely elevated by presence of the monoclonal paraprotein, potentially leading to erroneous classification of the clinical stage of the disease.

## 2. Case Presentation

Here, we report the case of a 47-year-old woman with a history of multinodular goiter, coronary bypass grafting, and an episode of mild anemia. She was referred to the outpatient clinic of internal medicine in our hospital by her general practitioner with headache, weight loss (five kg in four months), and a recurrence of mild anemia (hemoglobin level of 6.7 mmol/L; ref. 7.4–9.9 mmol/L). Laboratory investigations in hospital demonstrated low-normal hemoglobin levels (7.5 mmol/L), increased sedimentation rate (43 mm/h; ref. 1–20 mm/h), and (preexisting) mildly reduced kidney function (GFR-CKD-epi of 67 ml/min/1.73 m^2^; ref. > 90 ml/min/1.73 m^2^). Blood count did not show any abnormalities. Total plasma protein was elevated (87 g/L; ref. 57–82 g/L) and protein electrophoresis demonstrated a monoclonal paraprotein, type IgG kappa, at a concentration of 32 g/L. The presence of the monoclonal paraprotein raised the suspicion of multiple myeloma, which was confirmed by 50% monoclonal plasma cells in a bone marrow biopsy. For distinction between smouldering myeloma and myeloma (requiring therapy) [1], initial workup included laboratory tests investigating myeloma-defining events (CRAB criteria); both the criteria for anemia (Hb < 6.2 mmol/L) and for renal insufficiency (GFR-CKD-epi < 40 ml/min/1.73 m^2^) were not met [1]. However, plasma calcium measurement (by means of plasma total calcium measurement) was elevated, at 2.90 mmol/L (ref. 2.18–2.60 mmol/L), indicating hypercalcemia, potentially marking a myeloma-defining event. CT imaging of the axial skeleton did not show any presence of osteolytic lesions. Due to emerging pelvic pain, in addition, a CT vertebral column and pelvis was performed, which also did not show any presence of osteolytic lesions. Follow-up laboratory investigations into calcium homeostasis demonstrated normal levels of vitamin D (25-OH vitamin D, 136 nmol/L; ref. > 50 nmol/L), albumin (34 g/L; ref. 34–50 g/L), and parathyroid hormone (PTH) (4.8 pmol/L; ref. 2.0–9.3 pmol/L). Surprisingly, the plasma ionized calcium level was normal (1.18 mmol/L; ref. 1.10–1.30 mmol/L). Clinically, the patient did not show any symptoms related to hypercalcemia or to the high paraprotein level (e.g., hyperviscosity syndrome). Due to the counterintuitive and discrepant results of plasma ionized and total calcium levels in the absence of clinical evidence for bone lesions, the laboratory specialist was consulted, who typed this case as a (potential) case of pseudohypercalcemia.

**Figure 1:**
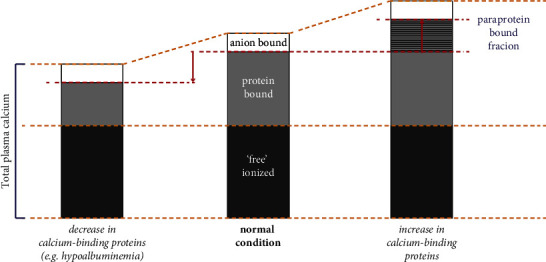
Impact of calcium-binding proteins on overall calcium distribution in plasma. In normal conditions (middle), approximately 50% of the calcium in blood is present as free ions, termed “ionized calcium”; about 40% of the calcium is bound to proteins (e.g. albumin); and the remainder of calcium is bound to anions, such as citrate and phosphate. The balance between these fractions is dependent on pH, the concentration of calcium-binding proteins, and the concentration of involved anions. Disruptions in this balance can result in temporary shifts in the fraction size, for instance, due to changes in the concentration of calcium-binding proteins, either due to loss of albumin (hypoalbuminemia), or by additional presence of a calcium-binding protein. Homeostatic mechanisms will respond to keep the (biologically important) ionized calcium fraction constant. As a result, the total calcium is either decreased or increased, leading to pseudohypocalcemia (left): a combination of decreased total calcium and normal ionized calcium (e.g., caused by hypoalbuminemia) or pseudohypercalcemia (right): a combination of increased total calcium and normal ionized calcium (caused by a larger protein-bound fraction, e.g., due to the presence of a calcium-binding paraprotein).

In blood, calcium is present in the following three distinct fractions: “free” ionized calcium (the biologically active fraction), bound to protein (such as albumin), and complexed to anions such as citrate, phosphate, and lactate (Figure 1) [2]. This balance can be disrupted and can lead to acute and temporary but also stable and persistent shifts in blood calcium fractions, leading to discrepancies between total plasma calcium and plasma ionized calcium measurements. In our case, total plasma calcium was elevated and the plasma ionized calcium level was normal, a phenomenon termed *pseudohypercalcemia*. It was suspected that this pseudohypercalcemia was caused by calcium binding to the monoclonal paraprotein present in the blood of the patient (i.e., an increased protein-bound calcium fraction). To investigate this, the patient's plasma (obtained at two different time points, two months apart) was analyzed further.

**Figure 2:**
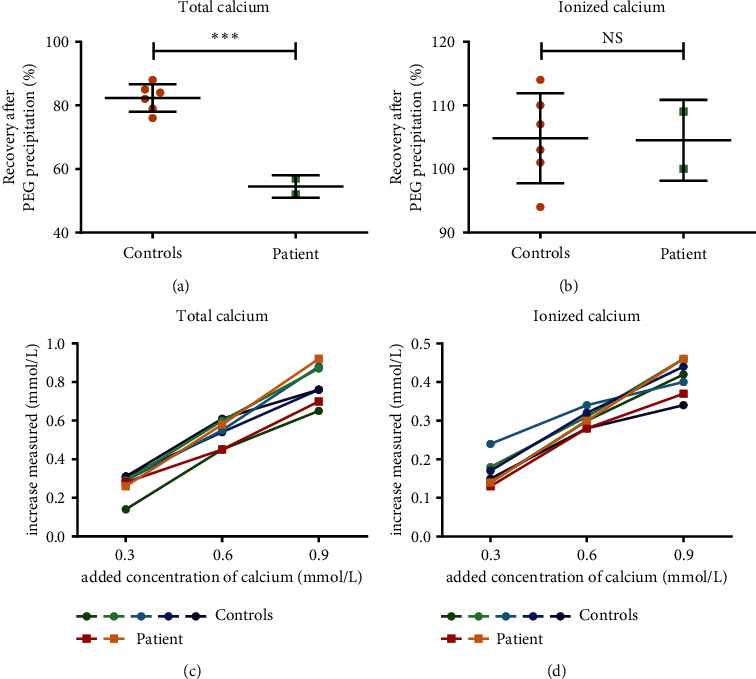
A calcium-binding plasma protein causes pseudohypercalcemia in the patient of interest, acting as a fully exploited calcium-binding reservoir. Recovery for total calcium (a) and recovery for ionized calcium (b) in plasma of the patient of interest (two different samples, two time points, and two months apart) and six controls with normal to high total calcium. Recovery is defined as the post-PEG treatment concentration of calcium as a percentage of the pretreatment calcium concentration (total calcium in (a) and ionized calcium in (b)). Bars represent the mean with SD. Statistical analysis (unpaired *t*-test) indicated a significant difference between patient and controls (^∗∗∗^*p*  <  0.05) for total calcium (a) but no significant difference between patient and controls for ionized calcium (NS) (b). Increase in total calcium levels (c) and increase in ionized calcium levels (d) in plasma of the patient of interest (two different samples, two time points, and two months apart) and five controls after addition of 0, 0.3, 0.6, and 0.9 M calcium. Control samples were available plasma samples from five persons (different from the six controls in (a) and (b)), selected based on total calcium values, albumin values, and total protein levels within reference interval and kidney function with GFR > 70 ml/min/1.73 m^2^.

First, precipitation of plasma proteins was performed with polyethylene glycol (PEG) precipitation, a widely used method to precipitate and detect (interfering) plasma proteins such as macroenzymes and immunoglobulins [3, 4]. Total calcium and ionized calcium levels were measured in the plasma of the patient and in the plasma of controls before and after PEG precipitation. Resulting recoveries for total calcium and ionized calcium (Figures 2(a) and 2(b)) clearly show that for our patient, the total calcium after PEG precipitation was substantially and significantly decreased (recovery of 52% and 57%) compared to total calcium in controls (recovery of on average 82%). PEG precipitation did not have any effect on ionized calcium levels; recovery was around 100% for all tested samples.

This means that a substantial portion of calcium present in the plasma of our patient (an estimated 0.9 mmol/L) was precipitated together with proteins precipitated by PEG. Since the monoclonal paraprotein was present in the patient's plasma at a concentration of 32 g/L (≈0.2 mmol/L) and was the most dominant protein present next to albumin, the additional protein binding capacity of the patient's plasma was attributed to the IgG kappa paraprotein.

Second, to investigate the calcium buffering capacity of this paraprotein, a calcium addition experiment was performed. A CaCl_2_ solution was added to the plasma of the patient and plasma of controls to increase the calcium concentration with 0.3 M, 0.6 M an 0.9 M respectively. After addition, both total and ionized calcium were measured. Surprisingly, similar calcium increases were observed in patient and control plasma upon addition of the calcium-containing solution, both in total and ionized calcium (Figures 2(c) and 2(d)), indicating that the extra calcium-binding capacity of the monoclonal protein was already maximally used.

Taken together, we conclude from these experiments that the monoclonal paraprotein IgG kappa present in the plasma of the patient binds calcium, with a capacity that was maximally used. This phenomenon leads to pseudohypercalcemia, explaining the discrepancy between ionized and total calcium levels (measured on multiple occasions) and the normal levels of vitamin D and PTH in the plasma of the patient.

**Figure 3:**
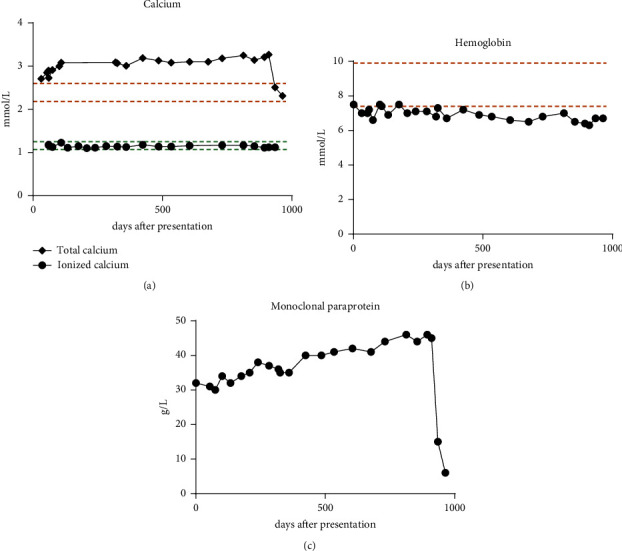
Calcium, hemoglobin and monoclonal protein levels in blood during patient follow-up and treatment. Total calcium and ionized calcium levels (a), hemoglobin levels, (b) and monoclonal paraprotein levels, and (c) in the patient's blood during follow-up and treatment over time, starting from the date of presentation. In (a), upper and lower limits of the reference interval for total calcium and ionized calcium are indicated in orange and in green, respectively. In (b), upper and lower limits of the reference interval of hemoglobin are indicated in orange.

These results were considered in the context of anamnesis, imaging, and other laboratory findings. In combination with the absence of myeloma-defining events (CRAB criteria), the (preliminary) diagnosis of smouldering myeloma was made. Since the patient did have not any physical complaints attributable to the smouldering myeloma, a wait-and-see policy was adopted. Plasma calcium parameters, hemoglobin level, and monoclonal paraprotein level were monitored over time (Figure 3). While total calcium levels were consistently high and above the reference interval, ionized calcium levels remained within the normal range, i.e., pseudohypercalcemia persisted (Figure 3(a)). Hemoglobin levels continued to be below the reference interval, only minimally declining after more than one year since presentation (Figure 3(b)). The monoclonal paraprotein steadily increased over time (Figure 3(c)). The patient's skeletal state was evaluated multiple times, with a CT of the axial skeleton. No evidence of osteolytic lesions was found up until 2.5 years after presentation, when endosteal scalloping and minimal punched out lesions were seen in all extremities. Soon thereafter (after 911 days since presentation), treatment with daratumumab and a combination of bortezomib, thalidomide, and dexamethasone was started, with an autologous stem cell transplantation trajectory planned afterwards. During the first cycle of treatment, the paraprotein level dropped from 45 to 15 g/L within four weeks (Figure 3(c)), with no significant changes in albumin levels or kidney function (data not shown). Within the same timeframe, the plasma total calcium level gradually dropped from 3.27 mmol/L to 2.51 mmol/L (which is within the normal range), whereas ionized calcium levels continued to be within the normal range (Figure 3(a)). These results confirm the role of calcium binding by the paraprotein in this case of pseudohypercalcemia and underline the additional value of ionized calcium measurement in diagnosis and follow-up of monoclonal gammopathies.

## 3. Discussion

In this case report, we demonstrate that pseudohypercalcemia due to a calcium-binding paraprotein is the cause of elevated total calcium levels in a patient with myeloma. The first case of pseudohypercalcemia due to a calcium-binding paraprotein was already identified as early as 1973 by Lindgarde and Zettervall [5]. Since then, several cases have been described, including calcium-binding IgG lambda, IgG kappa, and IgA paraproteins in multiple myeloma [6–8] but also calcium-binding IgM paraproteins in Waldenström's macroglobulinemia [9, 10]. In most reports, the phenomenon of pseudohypercalcemia was discovered in patients newly diagnosed with monoclonal gammopathies (with or without osteolytic lesions), but Jaffe and colleagues reported a case in which the patient had been already diagnosed with multiple myeloma for six years, exhibiting asymptomatic hypercalcemia resistant to treatment, prior to recognition as a case of pseudohypercalcemia [11].

Some early myeloma case report studies isolated the calcium-binding paraprotein (mostly IgG) and investigated its properties [5–7, 11–15]. Lindgarde and Zettervall were the first to investigate the calcium-binding IgG lambda paraprotein in their case patient with newly diagnosed multiple myeloma and asymptomatic hypercalcemia [5, 12, 13]. Using equilibrium dialysis and gel electrophoresis, they showed that specifically the paraprotein bound calcium (the patient's polyclonal IgG did not) [5, 12, 13]. Furthermore, they identified two independent calcium-binding sites in the paraprotein, located in the Fab region of the immunoglobulin, with a higher calcium-binding affinity than albumin [5, 12, 13].

Other studies later showed similar results; specifically, the patient's paraprotein was the calcium-binding protein (either the intact paraprotein or sometimes, when isolated, the light chain but never the heavy chain); the polyclonal IgG or IgA of the same patient could not bind calcium and neither did paraproteins of other tested patients [5, 11, 14, 15]. Two of these studies also found the calcium-binding sites to be located in the Fab region in their calcium-binding IgG kappa paraprotein, suggesting that the calcium-binding property may be related to the antigen-binding site of the immunoglobulin [6, 14]. The reported number of calcium ions bound per IgG molecule varied from 1.5 to 4, which approximates the ratio found in our case (0.9 mmol calcium per 0.2 mmol paraprotein) [5, 6, 11]. In fairly, all cases with calcium-binding IgG paraproteins trends in total calcium measurements followed trends in paraprotein levels, whether responsive or unresponsive to myeloma treatment [5, 15, 16].

Next to IgG paraproteins binding calcium, case reports with IgA and IgM paraproteins causing pseudohypercalcemia have also been described [7, 9, 17–19]. Pearce and colleagues isolated the IgA kappa paraprotein in their patient with pseudohypercalcemia and found multiple calcium-binding IgA fractions in gel electrophoresis, suggesting multiple polymeric forms of this IgA paraprotein [7]. Side et al. reported a patient with Waldenström's macroglobulinemia and a calcium-binding IgM kappa, isolating and investigating the IgM paraprotein fraction in the patient's serum; the paraprotein fraction of the case patient contained calcium upon extraction, and gel mobility of the paraprotein was affected by calcium, whereas gel mobility of IgM paraproteins of three other patients was unaffected [9]. In their case patient, there was a significant correlation between serum paraprotein concentration and total calcium levels (corrected for albumin) measured at various time points over a period of 20 months [9]. Multiple other case reports have reported pseudohypercalcemia in patients with Waldenström's macroglobulinemia but reported assay interference by the IgM paraprotein as the probable mechanism of cause [10, 17–20]. In most of these cases, total calcium measured with the Arsenazo III method was elevated, but calcium measurements using a different method (like atomic absorption spectrophotometry) yielded surprisingly normal results [10, 17–19]. These reports only provide indirect evidence for interference, as an underlying mechanism for interference by the paraprotein in the Arsenazo III method was not described. Direct analytical interference seems unlikely since the Arsenazo III method is a colorimetric detection method and immunoglobulins are colorless. Alternatively, the discrepancy between total calcium results obtained with the different methods may be explained by a calcium detection problem in atomic absorption spectrophotometry, where the paraprotein bound calcium fraction may stay undetected due to the tighter binding to the paraprotein. In our case, total calcium was also measured with the Arsenazo III method but was not measured with a different method. Another report suggesting assay interference by an IgM paraprotein used trichloroacetic acid (TCA; precipitating denatured proteins) to show that part of the elevated total calcium level measured in their patient with Waldenström's macroglobulinemia could be explained by assay interference [20]. However, TCA may strongly influence the pH of the plasma sample and hence influence the detection assay used to measure total calcium.

Next to pseudohypercalcemia in monoclonal gammopathy, a single case report interestingly described pseudohypercalcemia in a patient with mixed cryoglobulinemia as a complication of Sjögren's syndrome [21]. Although the authors hypothesize that calcium is needed for the cryoglobulines to precipitate to form cryocomplexes with their antigen IgG, extraction and in-depth characterization of the calcium-binding cryoglobulines were not performed [21].

Multiple case reports of patients with pseudohypercalcemia due to a calcium-binding paraprotein describe treatment of the patient for hypercalcemia. Treatment was most often initiated in the presence of osteolytic lesions even though the patient did not suffer from any hypercalcemia-related clinical symptoms [8, 11, 15, 16, 22–24]. Hypercalcemia treatment was started because of unawareness of the pseudohypercalcemia since ionized calcium was not (initially) measured [8, 11, 15, 16, 22–24]. Multiple treatment options for hypercalcemia were deployed (e.g., hydration, steroids, furosemide, mithramycin, bisphosphonates, and plasmapheresis) and in all cases, treatment did not lead to correction of elevated total calcium levels [11, 15, 16, 24]. In one unresponsive case, when treatment was stopped, total calcium continued to be elevated for at least three months without any clinical symptoms of hypercalcemia [15]. Erroneous initiation to “correct” the elevated total calcium level in pseudohypercalcemia may disturb the existing balance in calcium homeostasis to such a degree that the patient may develop hypocalcemia. The physiological response to this medically induced hypocalcemia will result in elevated PTH levels, which may be mistaken for primary hyperparathyroidism. This could lead to unnecessary diagnostic follow-up tests and imaging as described by two cases reported by Ashrafi et al. and Talha et al. [8, 22]. In a patient with pseudohypercalcemia and concurrent acute kidney injury, unjustly treatment of hypercalcemia during hospital admission even led to (transient) hypophosphatemia [23].

In conclusion, despite various case reports described in literature, the phenomenon of pseudohypercalcemia due to calcium binding to a monoclonal paraprotein remains an underrecognized condition among hematologists, as illustrated in our case. We plead for including ionized calcium measurements in case of diagnosis of monoclonal paraprotein disorders (as also advised by others [7, 8, 17–20, 22–25] and mentioned in recent guidelines for myeloma care [26]) in order to identify these cases at an early stage and prevent erroneous treatment and classification, especially when hypercalcemia (by means of elevated total calcium levels) is the only myeloma-defining event observed.

## Data Availability

The data that support the findings of this study are available from the corresponding author upon reasonable request. The data are not publicly available due to privacy restrictions.
